# Cellular Immune Response to COVID-19 and Potential Immune Modulators

**DOI:** 10.3389/fimmu.2021.646333

**Published:** 2021-04-30

**Authors:** Xi Zhou, Qing Ye

**Affiliations:** National Clinical Research Center for Child Health, National Children’s Regional Medical Center, The Children’s Hospital, Zhejiang University School of Medicine, Hangzhou, China

**Keywords:** COVID-19, SARS-COV-2, clinical outcome, cellular immune response, potential immune modulators

## Abstract

Coronavirus disease 2019 (COVID-19) is a respiratory infectious disease caused by severe acute respiratory syndrome coronavirus 2 (SARS-CoV-2). Droplets and contacts serve as the main route of transmission of SARS-CoV-2. The characteristic of the disease is rather heterogeneous, ranging from no symptoms to critical illness. The factors associated with the outcome of COVID-19 have not been completely characterized to date. Inspired by previous studies on the relevance of infectious diseases, viral and host factors related to clinical outcomes have been identified. The severity of COVID-19 is mainly related to host factors, especially cellular immune responses in patients. Patients with mild COVID-19 and improved patients with severe COVID-19 exhibit a normal immune response to effectively eliminate the virus. The immune response in patients with fatal severe COVID-19 includes three stages: normal or hypofunction, hyperactivation, and anergy. Eventually, the patients were unable to resist viral infection and died. Based on our understanding of the kinetics of immune responses during COVID-19, we suggest that type I interferon (IFN) could be administered to patients with severe COVID-19 in the hypofunctional stage, intravenous immunoglobulin (IVIG) and glucocorticoid therapy could be administered in the immune hyperactivation stage. In addition, low molecular weight heparin (LMWH) anticoagulation therapy and anti-infective therapy with antibiotics are recommended in the hyperactivation stage.

## Introduction

The ongoing outbreak of the coronavirus 2019 (COVID-19) caused by severe acute respiratory syndrome coronavirus 2 (SARS-CoV-2) has brought an unprecedented global health crisis (1–4). Droplet and contact transmission are the most common modes of transmission of SARS-CoV-2 (5). The disease’s characteristic is rather heterogeneous, ranging from no symptoms to critical illness, with 10%-20% symptomatic patients at considerable risk of fatality (2, 6, 7). Critical illness includes acute respiratory distress syndrome, septic shock, refractory metabolic acidosis, coagulopathies, dysfunction, and multiple organ failure, including heart, liver, kidney, and brain (8–13). Older age, male sex, and comorbidities have been associated with worse outcomes (14–16). In diseases caused by viral infections, viruses and hosts can contribute to disease heterogeneity. Studies have found that SARS-CoV-2 has limited genetic variation and stable evolution (17, 18), suggesting that viral genetic variation and evolution might contribute to infectivity and fatality (19, 20). However, not so much correlation is noted to the heterogeneity of COVID-19 (17, 21–23). Numerous studies have demonstrated that the severity and outcomes are closely related to hosts’ immune responses (22–27). The innate immune system with monocytes, granulocytes, dendritic cells (DCs), natural killer (NK) cells, and adaptive immune system with T and B lymphocytes are required to defend against SARS-CoV-2. Patients with severe COVID-19 exhibit lymphopenia with reduction in CD4+ and CD8+ T cells, lymphocyte activation and dysfunction, an increase in circulating neutrophils with the appearance of circulating neutrophil precursors, dysfunction of classical monocytes and loss of non-classical monocytes, reduced abundance and dysfunction of DCs and NK cells (22, 27, 28). Systemic inflammatory cytokine levels, especially interleukin IL-6 and IL-1 cytokines (29), are increased. In contrast, the interferon response is slower, and immunoglobulin G (IgG) and total antibody levels are increased (24, 30). Immune disorders are common in severe infections and sepsis and are characterized by developing a high inflammation state to immunosuppression. A similar mechanism has been proposed for severe COVID-19 (24, 31, 32). Due to the lack of specific antiviral drugs, the body’s immune response is a crucial factor affecting disease progression and prognosis. Therefore, a better understanding of the cellular immune response during the progression from mild disease to potentially fatal COVID-19 is crucial for developing diagnostic markers and strategies for the therapy of COVID-19.

In a SARS-CoV-2 infection, the activation, recruitment, and resolution of the antiviral immune response involve a highly organized cellular and molecular cascade. These cascades tightly regulate the balance between virus elimination and immune damage. During virus infection, multiple innate immune recognition mechanisms monitor and defend against viruses (33). Within a few hours, the innate immune system sends out a rapid antiviral response through type I/III IFN (34), cytokines (such as IL-1, IL-18, and IL-6), and chemokines (such as CCL2 and CCL7) to inhibit virus replication. Then, adaptive immunity is activated. T lymphocytes play a crucial role in virus clearance after virus infection, whereas humoral immunity mainly plays a role by producing antibodies and neutralizing viruses. T lymphocytes directly dissolve and destroy infected cells to eliminate viruses and secrete cytokines to enhance T lymphocytes’ immune response and other immunocompetent cells, such as macrophages and B lymphocytes. Then, the body downregulates innate immunity to avoid nonspecific damage to the host (35). When pathogens are eliminated, innate immune cells (such as macrophages and regulatory DCs) and adaptive regulatory cell types (such as regulatory T cells and B cells) also contribute to the resolution of inflammation (36).

**Figure 1:**
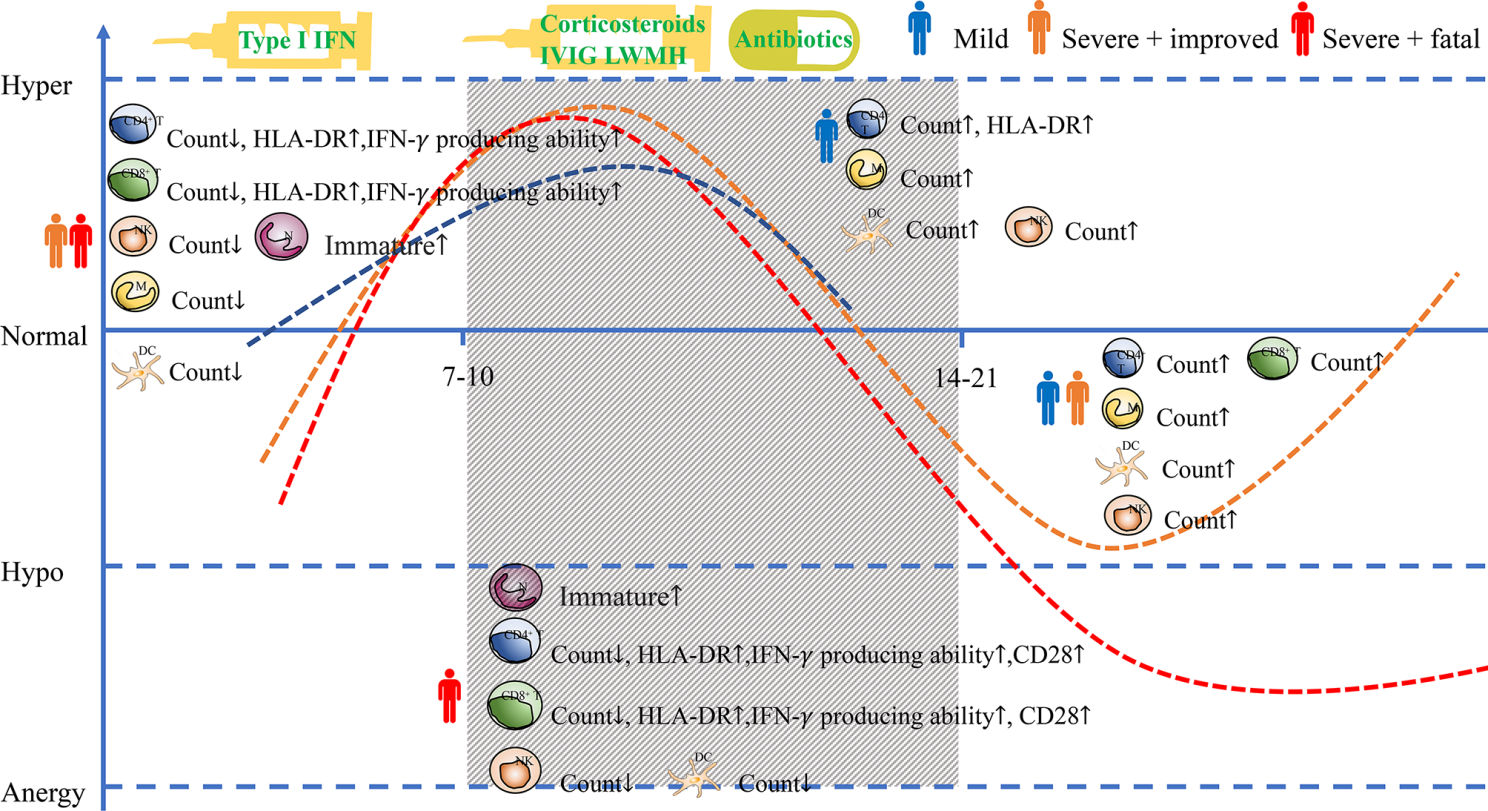
Cellular immune responses to SARS-CoV-2. The simulated diagram shows the kinetics of immune responses in COVID-19 patients with different clinical outcomes and the treatment in different stages. (↑ or ↓ means that compared with the result in the early stage, the number or activation of immune cells in described stage is increased or decreased) NK, nature killer cell; M, monocyte; N, neutrophil; DC, dendritic cell.

Based on previous studies on the Middle East respiratory syndrome (MERS), severe acute respiratory syndrome (SARS), and other coronavirus infections and clinical observations in COVID-19 patients (37, 38), the course of COVID-19 can be roughly divided into three stages: the first stage (the period up to 7-10 days after onset of symptoms,0 days to 7-10 days), the second stage (the period from 7-10 to 14-21 days after onset of symptoms) and the third stage (the period from 14-21 days or more after onset of symptoms). In the first stage, the patient is infected with the virus and develops no symptoms or influenza-like symptoms from mild to moderate, like fever, dry cough, and fatigue (3, 39). In this initial stage, the virus can be detected by polymerase chain reaction (PCR) analysis, and some asymptomatic infected people will transmit the virus to others (37). The virus can be suppressed and enter the recovery phase if the patients’ immune function is effective. However, suppose the patient is immune dysfunctional depending on age, gender, comorbidities, or other unknown factors. In that case, the virus cannot be suppressed effectively, and then the patients will progress to a severe phase. It was reported that the median time from the onset of symptoms to acute respiratory distress syndrome (ARDS) of COVID-19 patients is approximately eight days (40). After entering the second stage, patients with mild COVID-19 have basically recovered, whereas patients with non-mild illnesses worsen 7-10 days after the onset of symptoms. Chest imaging shows multiple ground glass shadows and infiltration shadows in both lungs (41). The patient presents dyspnea and/or severe hypoxemia. In the second stage, severe conditions can quickly progress to acute respiratory distress syndrome, septic shock, difficulty correcting metabolic acidosis, coagulation dysfunction, and multiple organ failure, including heart, brain, lung, liver, and kidney (42). Some patients with severe COVID-19 could not overcome the virus infection, unfortunately, and eventually die (**Figure 1**).

## The First Stage: Normal or Hypofunction Immune Response

It was noted that the total number of white blood cells in peripheral blood was normal or decreased in the early stage of COVID-19 (42). T and B lymphocyte subsets are important components of the immune cell population and important indicators for detecting immune function. T lymphocytes are classified into two important subsets: CD3+ CD4+ T lymphocytes and CD3+ CD8+ T lymphocytes. CD4+ T cells can differentiate into a range of helper and effector cell types, have the ability to indicate B cells, assist CD8+ T cells, recruit innate cells, have direct antiviral activity, and promote tissue repair. CD8+ T cells can kill infected cells and affect the immune response’s activation (43, 44). As another important component of the immune cell population, B lymphocyte subsets play a role in humoral immunity by secreting antibodies. Contrary to the increase in peripheral blood lymphocytes in conventional viral infections, lymphocyte counts in COVID-19 patients decrease compared with healthy controls. Peripheral blood lymphocyte levels are lower in patients with severe COVID-19 than those with mild COVID-19. A greater decline is noted in deceased patients than those with severe COVID-19 who ultimately survived (45–47). The number of T lymphocytes (including CD4+ and CD8+) in severe patients is lower than that in mild patients. The number of T lymphocytes was lower in deceased patients than in survived patients (45, 47). The function of T cells is lower in severe patients than that in mild patients. The frequencies of HLA-DR+ and IFN-γ+ cells within CD4+ and CD8+ cells are lower in deceased patients than survived patients, which indicated that T cells’ function is lower in deceased patients than survived patients. The B lymphocyte count is decreased. Patients with severe COVID-19 have lower B cell counts than patients with mild COVID-19, but the cells are within the normal range (45–47). Besides, B lymphocytes function is in a normal or slightly low state. The obvious decline in peripheral blood T lymphocytes may due to a large amount of immune cell chemotaxis to the site of inflammation or direct T cell damage induced by the SARS-CoV-2. B cells’ humoral immunity mainly occurs through the production of antibodies and virus neutralization, so the damage to B cells is not as significant as that to T cells.

The innate immune system is the first line of defense against the invasion of SARS-CoV-2, and the circulating innate immune cells in the blood, including neutrophils, NK cells, monocytes, DCs, change due to the initial local respiratory SARS-CoV-2 infection. Circulating neutrophils were increased in severe COVID-19 (45, 48). DCs are the body’s most potent full-time antigen-presenting cells (APCs) that can efficiently ingest, process, and present antigens (49). Immature DCs exhibit strong migration capabilities. Mature DCs can effectively activate naive T cells, which are at the center of initiating, regulating, and maintaining the immune response (49). No significant changes were identified in the proportion of DCs in patients with mild COVID-19. However, in severe patients, the number of DCs was lower than in mild patients (50) and significantly lower in deaths than survivors (46). Plasmacytoid dendritic cells (pDCs), which secret IFN α, were mainly reduced in abundance and impaired in function (25, 51). SARS-CoV-2 may alter the migration of impaired DCs, so T cells cannot be effectively activated (52).Further, emergency myelopoiesis with immature and dysfunctional neutrophils presented in severe COVID-19 (45). The number of monocytes was not significantly different between mild and severe patients (47). Studies have reported that the number of NK cells decreases in the early stage of COVID-19 (24, 45). Zhifeng Deng et al. (47) found that severe patients had higher NK cell counts compared with mild patients at this stage, and similar findings were noted in the comparison between survived patients and decreased patients. This finding may be attributed to the fact that patients’ innate immune function gradually declines in the early stage of symptom onset.

In this stage, the immune response of patients with mild COVID-19 and survived patients with severe COVID-19 is in a normal or hypofunctional state. The immune response of patients with fatal severe COVID-19 is hypofunctional with the reduced number and dysfunction of CD4+ and CD8+ T cells. Age and gender are the major risk factors for severe and fatal COVID-19. The changes in the innate immune system of the elderly, which has a reduced abundance of DCs with reduced type I IFN production, may relate to unfavorable outcomes (52, 53). Besides, autoantibodies to type I IFN are preferentially in the elderly and male population.

At this stage, the patient’s symptoms are mild. Patients with mild symptoms should rest in bed at this time, strengthen supportive treatment, ensure adequate energy intake, and monitor disease’s development. Patients with severe and high-risk conditions are advised to use drugs with potential antiviral effects. Research on patients with severe COVID-19 shows that gender and age differences may relate to type I IFN defects (46). Drugs targets to type I IFN might improve early innate immune responses. Multiple clinical studies have been conducted administrating type I IFN using different routes of administration. These results suggest that type I IFN may benefit COVID-19 recovery (54–56). A retrospective study found that those receiving type I IFN had a significantly lower mortality rate at an early stage. In contrast, late interferon therapy increased mortality and delayed recovery (57). In conclusion, the use of type I IFN at the early stage of COVID-19 helps treat severe cases (58–62).

## The Second Stage (Immune Response: Hyperactivation)

At this stage, patients with mild illness basically recover. The number of Peripheral blood lymphocytes in mild patients, including T and B lymphocyte, gradually increase (45, 47). At this stage, the condition of non-mild patients is further aggravated. Compared with mild patients, severe patients’ peripheral blood lymphocytes were further decreased (46), especially T lymphocytes (43, 59). Compared with patients recovering from severe illness, the expression of HLA-DR and IFN-γ synthesis of CD4+ and CD8+ T cells was significantly increased in deceased patients, which indicates that cell activity was significantly increased, and the phenomenon of excessive activation appeared (41, 60, 61). In particular, due to the rapid activation of pathogenic CD4+ T cells, which induce the enhancement of the mononuclear macrophage system’s function. Release many inflammatory factors and cause a cytokine storm, which may cause systemic multiple organ failure and lead to patient death (46, 62). Besides, the proportion of Treg lymphocytes in patients with a fatal disease is significantly increased at this stage. Treg cells typically negatively regulate the immune response, which implies the outcome of patients’ immune system status with the severe fatal disease. In patients with severe disease who survived, at this stage, HLA-DR expression and IFN-γ synthesis of CD4+ T cells increase, indicating the increased activity of CD4+ T cells. In contrast, the activity of CD8+ T cells decreases, and the immune system is not overactivated in severe patients who survived. The proportion of Treg cells decreased. Patients with severe disease who recover exhibit a gradual and stable recovery of immune function at this stage, while patients with severe disease who died exhibit excessive activation of the immune response (46).

NK cells, DCs, monocytes, and neutrophils exhibit normal levels in mild COVID-19 patients at this stage. A significantly higher proportion of granulocytes was observed in severe patients versus mild patients. Besides, dysfunctional, immature neutrophils were release, and mature, partially activated neutrophils were increased in severe COVID-19 patients indicated emergency myelopoiesis (45). The activation of neutrophils and the formation of neutrophil extracellular trap (NET) are related to coagulopathy. In addition, in some cases, the hypercoagulable state can lead to cortical necrosis and irreversible kidney failure (63). The abundance of circulating DCs in severely ill patients has progressively reduced. The number of DCs in deceased patients is significantly reduced compared with that in patients with severe disease who survive (46, 50, 64). The reduced abundance and exhausting function of circulating NK cells were noticed in severe COVID-19 patients (65). Deceased patients showed a lower count of NK cells than survived patients (66). Further, the number of monocytes in the severe recovery group remained at a certain level or slowly increased with normal function, whereas the number of severe fatal patients decreased significantly. In addition, cell function markers, such as HLA-DR and CD45, decreased, and cell activity was reduced. The innate immunity of severe fatal patients gradually collapsed (46, 47).

At this time, the overall immune function of mild patients and patients with severe disease who survived is increased, and CD4+ and CD8+ T cell activity is enhanced. However, the CD8+ T cell count is reduced (chemotaxis to the inflammatory site). B cells differentiate into plasma cells in large numbers. The number and activity of innate immune cells increased. The immune function of patients with severe fatal disease at this stage is excessively hyperactive. The number of CD4+ T cells and CD8+ T cells is further reduced, but the activity is significantly increased, as demonstrated by significantly increased HLA-DR expression and IFN-γ synthesis. In addition, the number and activity of innate immune cells are reduced. As the older individuals are more likely to have uncoordinated adaptive immunity response with abnormal T cell responses, the elderly has a higher risk of death from COVID-19 (26, 67).

No specific antiviral treatment for COVID-19 is currently available, so treating this disease mainly involves symptomatic treatment and oxygen therapy. It is recommended to monitor inflammatory factors and lymphocyte subsets during onset. When T cells, B cells, inflammatory cytokines, and D-dimers exhibit the following trends, IVIG, glucocorticoids, and other treatments are administered. Peripheral blood T lymphocytes were significantly reduced compared with previous levels, but T cells continued to be activated. Inflammatory cytokines, such as IL-6, were significantly increased. Thus, glucocorticoids and IVIG are administered in the second stage. Regarding immune changes, the immune system of mild patients can clear the virus without the aid of IVIG. In patients with severe COVID-19, the number of immune cells continues to decrease. Administration of high-dose IVIG at this phase of disease deterioration will be associated with markedly reduced mortality, quicker normalization of inflammatory status, and improved clinical outcomes in COVID-19 (68, 69). In the early stage, the inflammatory response is controllable, and the use of glucocorticoids will prolong the clearance time of SARS-CoV-2. In contrast, when the patients are in an immunosuppressed state, and glucocorticoids will worsen the disease. The second stage that the immune response is hyperactivation, is the most suitable time to use glucocorticoids. Studies have found that people who are severely infected with COVID-19 receive glucocorticoid treatment, and their chances of death are reduced compared with those who do not receive the treatment. Acute glucocorticoids with 6 mg of dexamethasone (equivalent to 40 mg daily of prednisone) daily for up to 10 days can reduce mortality from 25.7% to 22.9%. However, chronic glucocorticoid use has been found to increase the risk of poor outcomes in patients with COVID-19 (55, 70, 71). Preliminary data suggested that glucocorticoid reduced the risk of acute kidney injury (AKI) needing kidney replacement therapy. Acute kidney injury is one of the complications of severe COVID-19 (72). The present study reported results of high-dose IVIG use in patients with severe COVID-19 (73). A multicenter retrospective study in China of high-dose intravenous immunoglobulin in severe COVID-19 showed high-dose IVIG (0.3-0.5g/kg/day for five days) administered in severe COVID-19 patients within 14 days of onset was linked to reduced 28-day mortality (74). Evidence shows that coagulation disorder is common in severe cases (75). LMWH anticoagulant therapy can be strongly recommended in this stage. A cohort study of Low molecular weight heparin among patients with COVID-19 showed that LMWH, given at prophylactic dosage, was associated with better clinical outcomes (76). COVID-19 patients may be at risk of infection with bacterial, fungal, and other pathogens, leading to increased morbidity and mortality (77). The most commonly detected coinfection is a bacterial infection (77, 78). A study indicated that 50% of patients who died from COVID-19 had bacterial pneumonia (79). It is prudent to use antibiotics for patients with severe COVID-19 (80).

## The Third Stage (Immune Response: Anergy)

After the first and second stages of the patient’s immune system’s response to the virus, the virus can be effectively suppressed if the patient’s immune function is effective. Thus, the patient enters the recovery period. Suppose the patient’s immune function is impaired. In that case, the patient’s immune system will enter a state of incompetence, and the patient will not be able to resist the infection and eventually die.

The number of immune cells in some critically ill patients gradually increased, and their function was gradually recovered. Thus, these patients entered the recovery stage. Compared with mild patients, the immune response of patients with the disease who recover is slower, and the virus clearance time is prolonged. Thus, it takes longer for these patients to recover. The number of lymphocytes, including T lymphocytes, continues to decrease in patients with death (47). Cell function of CD4+ T cells is low as manifested by decreased activating receptors and increased CD45RA and CD28 expression (46). The number of B cells, NK cells, monocytes, and DCs in severe fatal patients is reduced. The immune system of patients with fatal disease enters a state of immune anergy after hyperactivation and eventually cannot resist viral infection.

At this stage, the overall immune function of surviving mild and severe patients is gradually recovered. Persistent changes including subset distribution, cell division, and expression of activation/exhaustion of circulating CD4+ T and CD8+ T cells were observed in recovered COVID-19 patients compared with healthy controls for at least six months. At 1.3 months, the relative proportions of circulating CD4+ T cells decreased, while circulating CD8+ T cells increased, although both had returned to relative physiologic levels by 6.1 months (81). The immune function of severe fatal patients gradually transforms into an anergy state, as the number and function of CD4+ T cells, CD8+ T cells, NK cells, monocytes, and DCs is dramatically reduced.

The typical features of severely ill patients include excessive lung inflammation and sepsis. Patients typically require an intensive care unit. Unfortunately, most patients cannot overcome the infection and eventually die.

## Conclusions

In summary, patients with mild COVID-19 and severe COVID-19 who survive exhibit a normal immune response that progresses from a normal or hypofunctional state to improved immune response and is finally restored to the pre-infection level. In patients with fatal COVID-19, the immune response ranges from diminished function to overactivation, eventually to a weakened immune response, and ultimately to death. In the current treatment of severe COVID-19 patients, it is critical to control the increase in deaths. We recommend that patients start interferon therapy in the early stage of the disease. IVIG and glucocorticoid therapy should be administered in the middle of the disease when the immune response is hyperactive. Appropriate antibiotics and anticoagulation therapy in the stage in which the immune response is hyperactive can improve patients with severe COVID-19. More research on the cellular immune response in COVID-19 is needed to help us understand the pathogenesis and guide the disease’s treatment to improve the prognosis.

However, this article focuses on the immune response of the host. In fact, the outcome of the disease is determined by both the host’s immune response and viral factors. For COVID-19, a severe disease, it is difficult to achieve good efficacy through immune regulation alone. Virus-specific treatment is also essential. Treatments targeting viral replication, which includes antiviral therapies like remdesivir, passive antibody therapies like monoclonal antibodies, and antibodies elicited from previous infection or vaccination (82–84), are effective in the disease process and could be more effective in the initial stage of the disease (85, 86). In addition, given the complexity and heterogeneity of immune response among individuals, though it is conceptionally reasonable, it may be practically challenging to use the immune modulator without a clear picture of the basal level and response among individuals. For example, the treatments that work for certain people with defined dose, duration, and frequency may not work for other populations. Therefore, the application of immunomodulation should be considered individually.

## Author Contributions

XZ conceived and wrote the manuscript and prepared figures. QY contributed to the modification and revision of the manuscript. All authors contributed to the article and approved the submitted version.

## Funding

This study was supported by key project of provincial ministry co‐construction, Health science and Technology project plan of Zhejiang Province (WKJ‐ZJ‐2128) and Key Laboratory of Women′s Reproductive Health Research of Zhejiang Province, Hangzhou, Zhejiang Province, P.R. China (No. ZDFY2020‐RH‐0006).

## Conflict of Interest

The authors declare that the research was conducted in the absence of any commercial or financial relationships that could be construed as a potential conflict of interest.

## References

[1] Xu X Chen P Wang J Feng J Zhou H Li X. Evolution of the Novel Coronavirus From the Ongoing Wuhan Outbreak and Modeling of its Spike Protein for Risk of Human Transmission. Sci China Life Sci (2020) 63(3):457–60. 10.1007/s11427-020-1637-5 PMC7089049 32009228

[2] Zhou P Yang XL Wang XG Hu B Zhang L Zhang W. A Pneumonia Outbreak Associated With a New Coronavirus of Probable Bat Origin. Nature (2020) 579(7798):270–3. 10.1038/s41586-020-2012-7 PMC7095418 32015507

[3] Chan JF Yuan S Kok KH To KK Chu H Yang J. A Familial Cluster of Pneumonia Associated With the 2019 Novel Coronavirus Indicating Person-to-Person Transmission: A Study of a Family Cluster. Lancet (2020) 395(10223):514–23. 10.1016/S0140-6736(20)30154-9 PMC7159286 31986261

[4] Ye Q Wang B Mao J Fu J Shang S Shu Q. Epidemiological Analysis of COVID-19 and Practical Experience From China. J Med Virol (2020) 92(7):755–69. 10.1002/jmv.25813 PMC7228220 32237160

[5] Chu DK Akl EA Duda S Solo K Yaacoub S Schunemann HJ. Physical Distancing, Face Masks, and Eye Protection to Prevent Person-to-Person Transmission of SARS-CoV-2 and COVID-19: A Systematic Review and Meta-Analysis. Lancet (2020) 395(10242):1973–87. 10.1016/S0140-6736(20)31142-9 PMC7263814 32497510

[6] Huang C Wang Y Li X Ren L Zhao J Hu Y. Clinical Features of Patients Infected With 2019 Novel Coronavirus in Wuhan, China. Lancet (2020) 395(10223):497–506. 10.1016/S0140-6736(20)30183-5 31986264 PMC7159299

[7] Gupta A Madhavan MV Sehgal K Nair N Mahajan S Sehrawat TS. Extrapulmonary Manifestations of COVID-19. Nat Med (2020) 26(7):1017–32. 10.1038/s41591-020-0968-3 PMC11972613 32651579

[8] Wiersinga WJ Rhodes A Cheng AC Peacock SJ Prescott HC. Pathophysiology, Transmission, Diagnosis, and Treatment of Coronavirus Disease 2019 (Covid-19): A Review. JAMA (2020) 324(8):782–93. 10.1001/jama.2020.12839 32648899

[9] Ye Q Lai E Luft F Persson P Mao J. Sars-CoV-2 Effects on the Renin-Angiotensin-Aldosterone System, Therapeutic Implications. Acta Physiol (Oxford England) (2020) 231(4):e13608. 10.1111/apha.13608 PMC7883221 33350096

[10] Han X Ye Q. Kidney Involvement in COVID-19 and its Treatments. J Med Virol (2021) 93(3):1387–95. 10.1002/jmv.26653 33150973

[11] Ye Q Lu D Shang S Fu J Gong F Shu Q. Crosstalk Between Coronavirus Disease 2019 and Cardiovascular Disease and its Treatment. ESC Heart Fail (2020) 7(6):3464–72. 10.1002/ehf2.12960 PMC7754975 32935928

[12] Ye Q Wang B Zhang T Xu J Shang S. The Mechanism and Treatment of Gastrointestinal Symptoms in Patients With COVID-19. Am J Physiol Gastrointestinal liver Physiol (2020) 319(2):G245–G52. 10.1152/ajpgi.00148.2020 PMC7414235 32639848

[13] Tian D Ye Q. Hepatic Complications of COVID-19 and its Treatment. J Med Virol (2020) 92(10):1818–24. 10.1002/jmv.26036 PMC7280725 32437004

[14] Grasselli G Zangrillo A Zanella A Antonelli M Cabrini L Castelli A. Baseline Characteristics and Outcomes of 1591 Patients Infected With SARS-Cov-2 Admitted to ICUs of the Lombardy Region, Italy. JAMA (2020) 323(16):1574–81. 10.1001/jama.2020.5394 PMC7136855 32250385

[15] Richardson S Hirsch JS Narasimhan M Crawford JM McGinn T Davidson KW. Presenting Characteristics, Comorbidities, and Outcomes Among 5700 Patients Hospitalized With Covid-19 in the New York City Area. JAMA (2020) 323(20):2052–9. 10.1001/jama.2020.6775 PMC7177629 32320003

[16] Docherty AB Harrison EM Green CA Hardwick HE Pius R Norman L. Features of 20 133 UK Patients in Hospital With covid-19 Using the ISARIC Who Clinical Characterisation Protocol: Prospective Observational Cohort Study. BMJ (2020) 369:m1985. 10.1136/bmj.m1985 32444460 PMC7243036

[17] Zhang X Tan Y Ling Y Lu G Liu F Yi Z. Viral and Host Factors Related to the Clinical Outcome of COVID-19. Nature (2020) 583(7816):437–40. 10.1038/s41586-020-2355-0 32434211

[18] Oude Munnink BB Nieuwenhuijse DF Stein M O’Toole A Haverkate M Mollers M. Rapid SARS-CoV-2 Whole-Genome Sequencing and Analysis for Informed Public Health Decision-Making in the Netherlands. Nat Med (2020) 26(9):1405–10. 10.1038/s41591-020-0997-y 32678356

[19] Le Borgne P Solis M Severac F Merdji H Ruch Y Alame Intern K. Sars-CoV-2 Viral Load in Nasopharyngeal Swabs in the Emergency Department Does Not Predict COVID-19 Severity and Mortality. Acad Emerg Med (2021) 28(3):306–13. 10.1111/acem.14217 PMC8014851 33481307

[20] Burgess S Smith D Kenyon JC Gill D. Lightening the Viral Load to Lessen covid-19 Severity. BMJ (2020) 371:m4763. 10.1136/bmj.m4763 33303483 PMC7614497

[21] Cho RHW To ZWH Yeung ZWC Tso EYK Fung KSC Chau SKY. Covid-19 Viral Load in the Severity of and Recovery From Olfactory and Gustatory Dysfunction. Laryngoscope (2020) 130(11):2680–5. 10.1002/lary.29056 PMC7436903 32794209

[22] Schultze JL Aschenbrenner AC. Covid-19 and the Human Innate Immune System. Cell (2021) 184(7):1671–92. 10.1016/j.cell.2021.02.029 PMC7885626 33743212

[23] Chua RL Lukassen S Trump S Hennig BP Wendisch D Pott F. Covid-19 Severity Correlates With Airway Epithelium-Immune Cell Interactions Identified by Single-Cell Analysis. Nat Biotechnol (2020) 38(8):970–9. 10.1038/s41587-020-0602-4 32591762

[24] Giamarellos-Bourboulis EJ Netea MG Rovina N Akinosoglou K Antoniadou A Antonakos N. Complex Immune Dysregulation in COVID-19 Patients With Severe Respiratory Failure. Cell Host Microbe (2020) 27(6):992–1000.e3. 10.1016/j.chom.2020.04.009 32320677 PMC7172841

[25] Lucas C Wong P Klein J Castro TBR Silva J Sundaram M. Longitudinal Analyses Reveal Immunological Misfiring in Severe COVID-19. Nature (2020) 584(7821):463–9. 10.1038/s41586-020-2588-y PMC7477538 32717743

[26] Rydyznski Moderbacher C Ramirez SI Dan JM Grifoni A Hastie KM Weiskopf D. Antigen-Specific Adaptive Immunity to SARS-CoV-2 in Acute Covid-19 and Associations With Age and Disease Severity. Cell (2020) 183(4):996–1012.e19. 10.1016/j.cell.2020.09.038 33010815 PMC7494270

[27] Sette A Crotty S. Adaptive Immunity to SARS-CoV-2 and COVID-19. Cell (2021) 184(4):861–80. 10.1016/j.cell.2021.01.007 PMC7803150 33497610

[28] Yang L Liu S Liu J Zhang Z Wan X Huang B. Covid-19: Immunopathogenesis and Immunotherapeutics. Signal Transduct Target Ther (2020) 5(1):128. 10.1038/s41392-020-00243-2 32712629 PMC7381863

[29] Ye Q Wang B Mao J. The Pathogenesis and Treatment of the `Cytokine Storm’ in COVID-19. J Infect (2020) 80(6):607–13. 10.1016/j.jinf.2020.03.037 PMC7194613 32283152

[30] Chen G Wu D Guo W Cao Y Huang D Wang H. Clinical and Immunological Features of Severe and Moderate Coronavirus Disease 2019. J Clin Invest (2020) 130(5):2620–9. 10.1172/JCI137244 PMC7190990 32217835

[31] Remy KE Brakenridge SC Francois B Daix T Deutschman CS Monneret G. Immunotherapies for COVID-19: Lessons Learned From Sepsis. Lancet Respir Med (2020) 8(10):946–9. 10.1016/S2213-2600(20)30217-4 PMC7195015 32444269

[32] Ritchie AI Singanayagam A. Immunosuppression for Hyperinflammation in COVID-19: A Double-Edged Sword? Lancet (2020) 395(10230):1111. 10.1016/S0140-6736(20)30691-7 PMC7138169 32220278

[33] Koch J Steinle A Watzl C Mandelboim O. Activating Natural Cytotoxicity Receptors of Natural Killer Cells in Cancer and Infection. Trends Immunol (2013) 34(4):182–91. 10.1016/j.it.2013.01.003 23414611

[34] Stetson DB Medzhitov R. Type I Interferons in Host Defense. Immunity (2006) 25(3):373–81. 10.1016/j.immuni.2006.08.007 16979569

[35] Kim KD Zhao J Auh S Yang X Du P Tang H. Adaptive Immune Cells Temper Initial Innate Responses. Nat Med (2007) 13(10):1248–52. 10.1038/nm1633 PMC2435248 17891146

[36] Dorward DA Russell CD Um IH Elshani M Armstrong SD Penrice-Randal R. Tissue-Specific Immunopathology in Fatal Covid-19. Am J Respir Crit Care Med (2020) 203(2):192–201. 10.1164/rccm.202008-3265OC PMC7874430 33217246

[37] Dos Santos WG. Natural History of COVID-19 and Current Knowledge on Treatment Therapeutic Options. BioMed Pharmacother (2020) 129:110493. 10.1016/j.biopha.2020.110493 32768971 PMC7332915

[38] Lin L Lu L Cao W Li T. Hypothesis for Potential Pathogenesis of SARS-CoV-2 Infection-a Review of Immune Changes in Patients With Viral Pneumonia. Emerg Microbes Infect (2020) 9(1):727–32. 10.1080/22221751.2020.1746199 PMC7170333 32196410

[39] Kong WH Li Y Peng MW Kong DG Yang XB Wang L. Sars-CoV-2 Detection in Patients With Influenza-Like Illness. Nat Microbiol (2020) 5(5):675–8. 10.1038/s41564-020-0713-1 32265517

[40] Wang D Hu B Hu C Zhu F Liu X Zhang J. Clinical Characteristics of 138 Hospitalized Patients With 2019 Novel Coronavirus-Infected Pneumonia in Wuhan, China. JAMA (2020) 323(11):1061–9. 10.1001/jama.2020.1585 PMC7042881 32031570

[41] Yonggang Zhou BF Zheng X Wang D Zhao C qi Y Sun R. Aberrant Pathogenic GM-CSF+T Cells and Inflammatory CD14+CD16+monocytes in Severe Pulmonary Syndrome Patients of a New Coronavirus. Natl Sci Rev (2020) 7(6):998–1002. 10.1101/2020.02.12.945576 PMC7108005 34676125

[42] National Health Commission of the People sRoC. Covid-19’s Diagnosis and Treatment Plan (Trial Eighth Edition). Infect Dis Immun (2020) 01(01):E1-E1. 10.1097/01.ID9.0000733564.2178

[43] Liu J Wang L Feng Z Geng D Sun Y Yuan G. Dynamic Changes of Laboratory Parameters and Peripheral Blood Lymphocyte Subsets in Severe Fever With Thrombocytopenia Syndrome Patients. Int J Infect Dis (2017) 58:45–51. 10.1016/j.ijid.2017.02.017 28249810

[44] Li S Cai C Feng J Li X Wang Y Yang J. Peripheral T Lymphocyte Subset Imbalances in Children With Enterovirus 71-Induced Hand, Foot and Mouth Disease. Virus Res (2014) 180:84–91. 10.1016/j.virusres.2013.11.021 24316007

[45] Schulte-Schrepping J Reusch N Paclik D Bassler K Schlickeiser S Zhang B. Severe COVID-19 is Marked by a Dysregulated Myeloid Cell Compartment. Cell (2020) 182(6):1419–40.e23. 10.1016/j.cell.2020.08.001 32810438 PMC7405822

[46] Zhang Q Bastard P Liu Z Le Pen J Moncada-Velez M Chen J. Inborn Errors of Type I IFN Immunity in Patients With Life-Threatening COVID-19. Science (2020) 370(6515):eabd4570. 10.1126/science.abd4570 32972995 PMC7857407

[47] Deng Z Zhang M Zhu T Zhili N Liu Z Xiang R. Dynamic Changes in Peripheral Blood Lymphocyte Subsets in Adult Patients With COVID-19. Int J Infect Dis (2020) 98:353–8. 10.1016/j.ijid.2020.07.003 PMC7334931 32634585

[48] Qin C Zhou L Hu Z Zhang S Yang S Tao Y. Dysregulation of Immune Response in Patients With Coronavirus 2019 (Covid-19) in Wuhan, China. Clin Infect Dis (2020) 71(15):762–8. 10.1093/cid/ciaa248 PMC7108125 32161940

[49] Worbs T Hammerschmidt SI Forster R. Dendritic Cell Migration in Health and Disease. Nat Rev Immunol (2017) 17(1):30–48. 10.1038/nri.2016.116 27890914

[50] Lee JS Park S Jeong HW Ahn JY Choi SJ Lee H. Immunophenotyping of COVID-19 and Influenza Highlights the Role of Type I Interferons in Development of Severe COVID-19. Sci Immunol (2020) 5(49):eabd1554. 10.1126/sciimmunol.abd1554 32651212 PMC7402635

[51] Kuri-Cervantes L Pampena MB Meng W Rosenfeld AM Ittner CAG Weisman AR. Comprehensive Mapping of Immune Perturbations Associated With Severe COVID-19. Sci Immunol (2020) 5(49):eabd7114. 10.1126/sciimmunol.abd7114 32669287 PMC7402634

[52] Shaw AC Goldstein DR Montgomery RR. Age-Dependent Dysregulation of Innate Immunity. Nat Rev Immunol (2013) 13(12):875–87. 10.1038/nri3547 PMC4096436 24157572

[53] Bastard P Rosen LB Zhang Q Michailidis E Hoffmann HH Zhang Y. Autoantibodies Against Type I Ifns in Patients With Life-Threatening COVID-19. Science (2020) 370(6515):eabd4585. 10.1126/science.abd4585 32972996 PMC7857397

[54] Hung IF Lung KC Tso EY Liu R Chung TW Chu MY. Triple Combination of Interferon beta-1b, Lopinavir-Ritonavir, and Ribavirin in the Treatment of Patients Admitted to Hospital With COVID-19: An Open-Label, Randomised, Phase 2 Trial. Lancet (2020) 395(10238):1695–704. 10.1016/S0140-6736(20)31042-4 PMC7211500 32401715

[55] Xu X Han M Li T Sun W Wang D Fu B. Effective Treatment of Severe COVID-19 Patients With Tocilizumab. Proc Natl Acad Sci USA (2020) 117(20):10970–5. 10.1073/pnas.2005615117 PMC7245089 32350134

[56] Davoudi-Monfared E Rahmani H Khalili H Hajiabdolbaghi M Salehi M Abbasian L. A Randomized Clinical Trial of the Efficacy and Safety of Interferon beta-1a in Treatment of Severe Covid-19. Antimicrob Agents Chemother (2020) 64(9):e01061-20. 10.1128/AAC.01061-20 32661006 PMC7449227

[57] Wang N Zhan Y Zhu L Hou Z Liu F Song P. Retrospective Multicenter Cohort Study Shows Early Interferon Therapy is Associated With Favorable Clinical Responses in COVID-19 Patients. Cell Host Microbe (2020) 28(3):455–64.e2. 10.1016/j.chom.2020.07.005 32707096 PMC7368656

[58] Schreiber G. The Role of Type I Interferons in the Pathogenesis and Treatment of COVID-19. Front Immunol (2020) 11:595739. 10.3389/fimmu.2020.595739 33117408 PMC7561359

[59] Diao B Wang C Tan Y Chen X Liu Y Ning L. Reduction and Functional Exhaustion of T Cells in Patients With Coronavirus Disease 2019 (Covid-19). Front Immunol (2020) 11:827. 10.3389/fimmu.2020.00827 32425950 PMC7205903

[60] Ni L Ye F Cheng ML Feng Y Deng YQ Zhao H. Detection of SARS-CoV-2-Specific Humoral and Cellular Immunity in COVID-19 Convalescent Individuals. Immunity (2020) 52(6):971–7.e3. 10.1016/j.immuni.2020.04.023 32413330 PMC7196424

[61] Li CK Wu H Yan H Ma S Wang L Zhang M. T Cell Responses to Whole SARS Coronavirus in Humans. J Immunol (2008) 181(8):5490–500. 10.4049/jimmunol.181.8.5490 PMC2683413 18832706

[62] Xu Z Shi L Wang Y Zhang J Huang L Zhang C. Pathological Findings of COVID-19 Associated With Acute Respiratory Distress Syndrome. Lancet Respir Med (2020) 8(4):420–2. 10.1016/S2213-2600(20)30076-X PMC7164771 32085846

[63] Batlle D Soler MJ Sparks MA Hiremath S South AM Welling PA. Acute Kidney Injury in COVID-19: Emerging Evidence of a Distinct Pathophysiology. J Am Soc Nephrol (2020) 31(7):1380–3. 10.1681/ASN.2020040419 PMC7350999 32366514

[64] Wilk AJ Rustagi A Zhao NQ Roque J Martinez-Colon GJ McKechnie JL. A Single-Cell Atlas of the Peripheral Immune Response in Patients With Severe COVID-19. Nat Med (2020) 26(7):1070–6. 10.1038/s41591-020-0944-y PMC7382903 32514174

[65] Zheng M Gao Y Wang G Song G Liu S Sun D. Functional Exhaustion of Antiviral Lymphocytes in COVID-19 Patients. Cell Mol Immunol (2020) 17(5):533–5. 10.1038/s41423-020-0402-2 PMC7091858 32203188

[66] Wang F Hou H Yao Y Wu S Huang M Ran X. Systemically Comparing Host Immunity Between Survived and Deceased COVID-19 Patients. Cell Mol Immunol (2020) 17(8):875–7. 10.1038/s41423-020-0483-y PMC7295144 32541836

[67] Arunachalam PS Wimmers F Mok CKP Perera R Scott M Hagan T. Systems Biological Assessment of Immunity to Mild Versus Severe COVID-19 Infection in Humans. Science (2020) 369(6508):1210–20. 10.1126/science.abc6261 PMC7665312 32788292

[68] Nguyen AA Habiballah SB Platt CD Geha RS Chou JS McDonald DR. Immunoglobulins in the Treatment of COVID-19 Infection: Proceed With Caution! Clin Immunol (2020) 216:108459. 10.1016/j.clim.2020.108459 32418917 PMC7211658

[69] Gharebaghi N Nejadrahim R Mousavi SJ Sadat-Ebrahimi SR Hajizadeh R. The Use of Intravenous Immunoglobulin Gamma for the Treatment of Severe Coronavirus Disease 2019: A Randomized Placebo-Controlled Double-Blind Clinical Trial. BMC Infect Dis (2020) 20(1):786. 10.1186/s12879-020-05507-4 33087047 PMC7576972

[70] Robinson PC Morand E. Divergent Effects of Acute Versus Chronic Glucocorticoids in COVID-19. Lancet Rheumatol (2021) 3(3):e168–e70. 10.1016/S2665-9913(21)00005-9 PMC7833899 33521656

[71] Group RC Horby P Lim WS Emberson JR Mafham M Bell JL. Dexamethasone in Hospitalized Patients With Covid-19. N Engl J Med (2021) 384(8):693–704. 10.1056/NEJMoa2021436 32678530 PMC7383595

[72] Bruchfeld A. The COVID-19 Pandemic: Consequences for Nephrology. Nat Rev Nephrol (2021) 17(2):81–2. 10.1038/s41581-020-00381-4 PMC7703720 33257872

[73] Xie Y Cao S Dong H Li Q Chen E Zhang W. Effect of Regular Intravenous Immunoglobulin Therapy on Prognosis of Severe Pneumonia in Patients With COVID-19. J Infect (2020) 81(2):318–56. 10.1016/j.jinf.2020.03.044 PMC7151471 32283154

[74] Cao W Liu X Hong K Ma Z Zhang Y Lin L. High-Dose Intravenous Immunoglobulin in Severe Coronavirus Disease 2019: A Multicenter Retrospective Study in China. Front Immunol (2021) 12:627844. 10.3389/fimmu.2021.627844 33679771 PMC7933558

[75] Iba T Levy JH Levi M Thachil J. Coagulopathy in COVID-19. J Thromb Haemost (2020) 18(9):2103–9. 10.1111/jth.14975 PMC7323352 32558075

[76] Qin W Dong F Zhang Z Hu B Chen S Zhu Z. Low Molecular Weight Heparin and 28-Day Mortality Among Patients With Coronavirus Disease 2019: A Cohort Study in the Early Epidemic Era. Thromb Res (2021) 198:19–22. 10.1016/j.thromres.2020.11.020 33249247 PMC7681071

[77] Lansbury L Lim B Baskaran V Lim WS. Co-Infections in People With COVID-19: A Systematic Review and Meta-Analysis. J Infect (2020) 81(2):266–75. 10.1016/j.jinf.2020.05.046 PMC7255350 32473235

[78] Hughes S Troise O Donaldson H Mughal N Moore LSP. Bacterial and Fungal Coinfection Among Hospitalized Patients With COVID-19: A Retrospective Cohort Study in a UK Secondary-Care Setting. Clin Microbiol Infect (2020) 26(10):1395–9. 10.1016/j.cmi.2020.06.025 PMC7320692 32603803

[79] Zhou F Yu T Du R Fan G Liu Y Liu Z. Clinical Course and Risk Factors for Mortality of Adult Inpatients With COVID-19 in Wuhan, China: A Retrospective Cohort Study. Lancet (2020) 395(10229):1054–62. 10.1016/S0140-6736(20)30566-3 PMC7270627 32171076

[80] Lucien MAB Canarie MF Kilgore PE Jean-Denis G Fenelon N Pierre M. Antibiotics and Antimicrobial Resistance in the COVID-19 Era: Perspective From Resource-Limited Settings. Int J Infect Dis (2021) 104:250–4. 10.1016/j.ijid.2020.12.087 PMC7796801 33434666

[81] Breton G Mendoza P Hagglof T Oliveira TY Schaefer-Babajew D Gaebler C. Persistent Cellular Immunity to SARS-CoV-2 Infection. J Exp Med (2021) 218(4):e20202515. 10.1084/jem.20202515 33533915 PMC7845919

[82] Vabret N Britton GJ Gruber C Hegde S Kim J Kuksin M. Immunology of COVID-19: Current State of the Science. Immunity (2020) 52(6):910–41. 10.1016/j.immuni.2020.05.002 PMC7200337 32505227

[83] Meganck RM Baric RS. Developing Therapeutic Approaches for Twenty-First-Century Emerging Infectious Viral Diseases. Nat Med (2021) 27(3):401–10. 10.1038/s41591-021-01282-0 33723456

[84] Behrouzi B Araujo Campoverde MV Liang K Talbot HK Bogoch II McGeer A. Influenza Vaccination to Reduce Cardiovascular Morbidity and Mortality in Patients With COVID-19: Jacc State-of-the-Art Review. J Am Coll Cardiol (2020) 76(15):1777–94. 10.1016/j.jacc.2020.08.028 PMC7535809 33032740

[85] Asselah T Durantel D Pasmant E Lau G Schinazi RF. Covid-19: Discovery, Diagnostics and Drug Development. J Hepatol (2021) 74(1):168–84. 10.1016/j.jhep.2020.09.031 PMC7543767 33038433

[86] Attaway AH Scheraga RG Bhimraj A Biehl M Hatipoglu U. Severe covid-19 Pneumonia: Pathogenesis and Clinical Management. BMJ (2021) 372:n436. 10.1136/bmj.n436 33692022

